# In search of a marker of altered cerebrovascular function in hypertension: Analysis of the fractional amplitude of low-frequency fluctuations in UK Biobank resting state fMRI data

**DOI:** 10.1016/j.cccb.2023.100196

**Published:** 2023-12-19

**Authors:** Owen Bleddyn Woodward, Ian Driver, Emma Hart, Richard Wise

**Affiliations:** a Cardiff University Brain Research Imaging Centre, Cardiff, Wales, United Kingdom; b School of Physiology, Pharmacology & Neuroscience, University of Bristol, United Kingdom; c Department of Neuroscience, Imaging, and Clinical Sciences, University G. D'Annunzio of Chieti-Pescara, Chieti, Italy; d Institute for Advanced Biomedical Technologies (ITAB), University G. D'Annunzio of Chieti-Pescara, Chieti, Italy

**Keywords:** Cerebral blood flow, Hypertension, Cerebrovascular reactivity, Brainstem

## Abstract

The selfish brain mechanism proposes that in some patients with impaired cerebral blood flow (CBF) or cerebrovascular function, hypertension may develop as a compensatory mechanism that aims to maintain CBF by increasing systemic blood pressure through an increase in cardiovascular sympathetic tone. The amplitude of low frequency fluctuations (ALFF) in the resting state blood oxygenation level dependent (BOLD) functional MRI signal has been previously posited as an index of cerebrovascular reactivity.

We investigated whether regional fractional ALFF (fALFF) differs between 2054 hypertensives and 1724 normotensives using data from the UK Biobank dataset. Our primary hypothesis was that cerebrovascular function in the medulla and other regions involved in sympathetic regulation differs between hypertensives and normotensives, and that this is reflected by regional variations in fALFF.

There is a significant regional variation in fALFF (F(14) =1126.17, *p <* 2 × 10^−16^, partial η^2^ = 0.22), but this regional variation does not differ between hypertensives and normotensives (F(14) = 0.23, *p* = 0.99, partial η^2^ = 8 × 10^−5^).

Prospective longitudinal studies of cerebral haemodynamics in hypertensives and normotensives are required to further investigate the selfish brain mechanism.

## Highlights

• There is a significant regional variation in the fractional amplitude of low frequency fluctuations (fALFF) in the resting state BOLD signal in the brainstem and other parts of the central autonomic network but no association between regional fALFF and hypertension.
• fALFF in regions of the brain involved in sympathetic activity does not seem to have a specific association with hypertension.
• fALFF is unlikely to be useful as a specific distinguishing marker of cerebrovascular reactivity in the context of hypertension.

## 1 Introduction

Hypertension is highly prevalent [1] and is associated with multiple comorbidities including stroke, coronary artery disease and renal disease [2]. As of 2015, 874 million adults worldwide had a systolic blood pressure that met the National Institute for Health and Care Excellence's (NICE) stage 1 hypertension threshold [1]. Despite the high prevalence and high morbidity associated with hypertension, its aetiology is poorly understood [3].

As well as being subject to end organ damage from hypertension, the brain may play a causal role via the selfish brain mechanism, which proposes that in some people hypertension is a compensatory mechanism that aims to maintain cerebral blood flow (CBF) by increasing systemic blood pressure through an increase in cardiovascular sympathetic tone. Several subcortical regions are involved in the modulation of sympathetic nerve activity, including nuclei in the pons, midbrain, thalamus, hypothalamus, insula, amygdala and hippocampus [4], [5], [6], [7], but the medulla in particular houses several nuclei, including the rostral ventrolateral medulla (RVLM) and the nucleus tractus solitarii (NTS) [8], which are thought to play a central role in the autonomic regulation of the cardiovascular system [9,10]. According to the selfish brain mechanism, if CBF is compromised, increased neuronal activity in regions of the brain which modulate sympathetic nerve activity, such as the medulla, elevates cardiovascular sympathetic tone and subsequently increases systemic blood pressure to maintain cerebral perfusion.

The cause of this hypothesised reduction in CBF is uncertain. In animal models of human essential hypertension there is evidence of an increased expression of proinflammatory molecules localised to the microvascular endothelium of the NTS, such as junctional adhesion molecule 1 (JAM-1) and endothelial nitric oxide synthase (eNOS) [11]. Furthermore, Paton et al. have demonstrated that occlusion of caudal medullary draining veins reduces blood flow and oxygenation to the medulla of normotensive baroreceptor reflex dennervated rats, which induces a significant increase in their arterial blood pressure [11]. Thus, impairment of cerebrovascular function in the medulla, mediated by overexpression of proinflammatory molecules and coupled with dysfunctional baroreceptor mechanism, may lead to hypertension and could underpin the selfish brain mechanism. In hypertensive humans, there is evidence of an association between hypoplasia of the vertebral arteries (the main arterial supply to the brainstem) and a global reduction in CBF [12]. However, there is no direct evidence from human studies to support impaired cerebrovascular function specifically involving the medullary autonomic centres in the development of hypertension.

One index of cerebrovascular function in humans is cerebrovascular reactivity (CVR). CVR quantifies the change in CBF in response to given vascular stimulus. It is related to the responsiveness, tone and functional reserve of the vascular system and is often regarded as an important marker of the health of the cerebrovascular system. Impaired CVR has been associated with many diseases, including stroke and transient ischaemic attack (TIA) [13] and multiple sclerosis [14]. Assessment of CVR usually requires the administration of a vasoactive substance such as carbon dioxide or acetazolamide to modulate CBF. However, the amplitude of low frequency fluctuation (ALFF) in the resting-state BOLD signal is a potential surrogate marker of CVR. Golestani et al. have previously demonstrated a significant association in the motor and executive control networks between CVR, generated by measuring the CBF response to a hypercapnic challenge, and ALFF in the 0.008 – 0.09 Hz range [15]. Furthermore, Ni et al. have demonstrated a significant widespread correlation between CVR maps derived from resting-state BOLD data and ALFF in the 0.01 – 0.08 Hz range [16]. ALFF has previously been found to vary between various physiological and pathophysiological states. For example, Yang et al. found ALFF in the visual cortices to be significantly higher with the eyes open than with the eyes closed [17]. Other studies have suggested regional differences in ALFF between different diseases such as major depressive disorder [18], mesial temporal lobe epilepsy [19], Alzheimer's [20], and migraine [21]. ALFF is therefore an useful index of neurovascular function that can be readily derived from resting-state functional MRI (rsfMRI) data, and given the haemodynamic component of the BOLD signal and the evidence of an association between CVR and ALFF [15,16], it is reasonable to posit ALFF as a possible index of cerebrovascular reactivity (CVR).

**Fig. 1:**
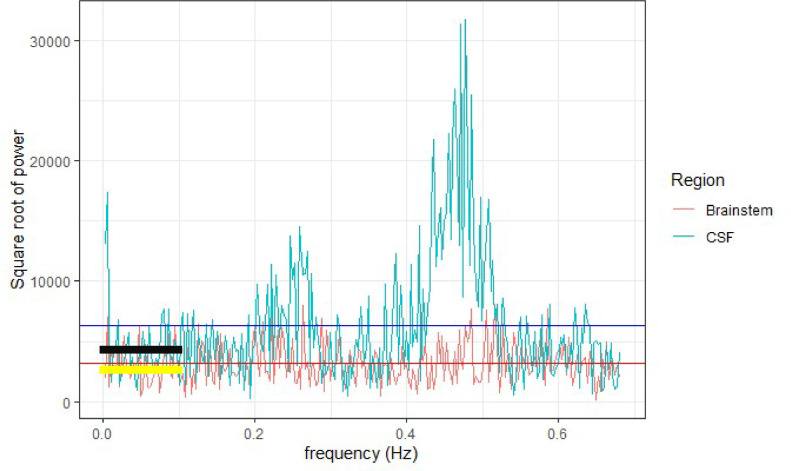
Brainstem (red) and CSF (cyan) voxel power spectrum. The power in the CSF voxel is higher across the entire frequency spectrum, but especially at higher frequencies. Horizontal blue line = mean power in CSF voxel, horizontal red line = mean power in brainstem voxel. The ALFF in the CSF voxel (black line) is higher than the ALFF in the brainstem (yellow line). However, the ratio of ALFF to the mean amplitude of the power spectrum (i.e. fractional ALFF) is higher in the brainstem than in the CSF. Therefore, CSF fALFF is suppressed relative to brainstem fALFF by the fractional correction. (For interpretation of the references to color in this figure legend, the reader is referred to the web version of this article.)

Fluctuations in the resting state BOLD signal due to physiological processes have been shown to contaminate ALFF measurements. High-powered fluctuations within the CSF spaces will dominate over lower-powered fluctuations in the brain parenchyma, reducing the sensitivity of the ALFF analysis [22] (see Fig. 1). The present analysis is especially concerned with investigating subcortical regions, including the brainstem, which are surrounded by CSF spaces, and therefore in this study the ratio of the power at each frequency to the mean power across the whole frequency spectrum was calculated. This ratio, known as fractional ALFF (fALFF) [22], suppresses contributions from the CSF spaces and large vessels, improving the sensitivity to detect variations in ALFF between brain regions (Fig. 1). We investigated differences in bandpass filtered fractional ALFF from rsfMRI data in a group of hypertensives compared to a group of normotensive controls, selected from the UK Biobank, focussing on regions of the brain involved in autonomic regulation. The selfish brain mechanism proposes that cerebrovascular abnormalities precipitate hypertension. However, hypertension is itself causally associated with pathological changes to small subcortical and cortical perforating cerebral blood vessels [23] leading to chronic cerebral microangiopathy [24]. Whilst a causal relationship between fALFF and hypertension cannot be established by this cross-sectional study, cerebrovascular disease caused by hypertension would be expected to affect the anterior and posterior circulation reasonably equally in a group-level analysis. If impaired cerebrovascular function specifically involving the posterior circulation (mediated by vertebral artery hypoplasia) or the medulla (mediated by, for example, localised brainstem inflammation) plays a causal role in the development of hypertension, a significant differential between CVR in the posterior circulation and/or medulla compared to the anterior circulation would be expected. Therefore, our primary hypothesis was that fALFF in the posterior circulation, and specifically the medulla, would be lower in hypertensives. An association between impaired medullary fALFF and hypertension would be consistent with the selfish brain mechanism, and such a finding would support the development of further longitudinal research to elucidate the direction of any such association.

## 2 Methods

UK Biobank is a large biomedical database containing genetic and health information on over 500,000 participants [25]. Approval was obtained from UK Biobank to download and analyse rsfMRI data.

International Statistical Classification of Diseases and Related Health Problems revision-10 (ICD-10) diagnostic codes from hospital inpatient records were used to classify participants as hypertensive or normotensive. ICD-10 coding explicitly differentiates essential and secondary hypertension and contains multiple sub-categories detailing additional diagnoses such as ‘hypertension with heart disease or heart failure’, and ‘hypertension secondary to renal disorders’. Only participants with essential hypertension were included.

Age [26], sex [27] and BMI [28] are all associated with blood pressure variability. To account for these potentially confounding variables, propensity score matching was performed using the R software package ‘MatchIt’ [29]. The propensity score describes the distribution of confounds within each participant [30]. For example, two subjects, one hypertensive and one normotensive, with similar propensity score will have similar values of age, sex and BMI. Propensity score matching can therefore be used to ensure that the normotensive and hypertensive groups are equivalent according to age, sex and BMI.

### 2.1 MRI analysis

UK Biobank rsfMRI data were acquired at three dedicated imaging centres, each equipped with identical 3T Siemens Skyra systems using a standard Siemens 32-channel receive head coil. rsfMRI parameters are as follows: Resolution: 2.4 mm isotropic, 88 × 88 × 64 matrix, 6 min duration (490 timepoints), TR: 0.735 s, TE: 39 ms, GE-EPI with x8 multislice acceleration, no iPAT, flip angle 52°, fat saturation. Further details of UK Biobank MRI protocols and acquisition parameters are available via Alfaro-Almagro et al. [31].

High resolution T1-weighted structural images (1 mm isotropic resolution) were also available from UK Biobank in pre-processed form [31]. This included tissue-type segmentation using FAST (FMRIB's Automated Segmentation Tool) [32], and co-registration to a MNI152 template in standard space using a combination of linear and non-linear transformations which were subsequently combined into a single structural-to-MNI non-linear warp field.

Generic pre-processing steps were applied to all rsfMRI data. Data were motion-corrected and brain extracted using FSL MCFLIRT and BET respectively. Spatial smoothing was applied with a kernel full-width half maximum (FWHM) equal to twice the voxel size of the functional data. Co-registration from each individual's subject space to their structural image was performed using FSL FLIRT. The resulting transform was combined with the structural-to-MNI non-linear warp field available from UK Biobank in order to define the transform from the functional dataset to the standard MNI152 template.

### 2.2 ALFF and fractional ALFF

Previous studies have investigated ALFF using various bandpass frequencies [33] but most of the literature has focused on a frequency range between 0.01 and 0.08 Hz. Recent work by Liu et al. [34] used the global mean rsfMRI time course, bandpass filtered, as a regressor in a general linear model (GLM) and investigated which frequency range of the rsfMRI time course led to rsCVR maps with the strongest correlation with CVR maps generated using a hypercapnic challenge. A frequency range of 0 to 0.1164 Hz was found to have the strongest correlation. Therefore, we initially selected the broadest frequency range (between 0 and 0.1164 Hz) suggested by the literature to maximise sensitivity to detect differences in CVR between hypertensives and normotensives. However, low-frequency fluctuations in heart rate [35] and systemic blood pressure [36] might contribute to ALFF in this frequency range. Therefore, to confirm that the results of the analysis in the 0 to 0.1164 Hz frequency range were not significantly affected by the above confounds, the same 2054 hypertensive and 1724 normotensive rsfMRI datasets were re-analysed using the narrower frequency band of 0.01 to 0.08 Hz.

**Fig. 2:**
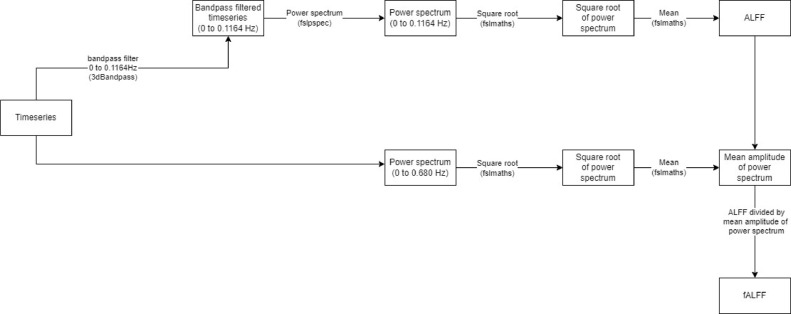
Outline of fALFF calculation between 0 and 0.01164 Hz. The same process was followed to calculate fALFF between 0.01 and 0.08 Hz.

Voxelwise fALFF was calculated as follows. Firstly, the linear trend in the data was removed (to address the possible confound of low frequency drifts in the MRI signal) then the data were filtered between the desired frequencies (low pass filter up to 0.1164 Hz in the initial analysis, and a bandpass filter between 0.01 and 0.08 Hz in the subsequent analysis). The voxelwise bandpass filtered data were transformed to voxelwise power spectra using *fslpspec*. The power of a frequency component is proportional to the square of the amplitude at that frequency. The square root of the voxelwise power spectrum data was obtained, and the mean was calculated to give the ALFF (Fig. 2).

The fractional ALFF correction was then applied as follows. The mean amplitude of the square root of the power spectra across the entire frequency range (0 to 0.680 Hz) of the rsfMRI signal was calculated (the upper frequency limit of the rsfMRI data is defined according to the Nyquist limit and the sampling rate of the rsfMRI acquisition (TR =  0.735 s)). The voxelwise mean ALFF maps were then divided within each participant by these maps to produce maps displaying voxelwise ALFF as a fraction of the mean amplitude of signal fluctuations across the entire rsfMRI frequency spectrum (fALFF).

Regional brainstem masks were generated by segmentation of the MNI152 template using FreeSurfer [37], a software tool that enables whole-brain segmentation. FreeSurfer segments the brainstem into three regions, the midbrain, pons and medulla. Harvard-Oxford cortical atlas was used to generate masks for the other regions of interest (amygdala, hippocampus, hypothalamus, thalamus, insula cortex (all of which are implicated in the modulation of autonomic activity [4–7]) as well as the visual cortex and frontal grey matter). These masks were used to determine mean regional fALFF values by applying them to fALFF z-score maps that had been transformed to MNI space.

### 2.3 Statistical analysis

Because we are interested in whether there is a differential between anterior and posterior circulation fALFF in hypertensives, we standardised against the anterior circulation ROI (frontal grey matter). If posterior circulation CVR is indeed impaired in the presence of hypertension, standardisation of fALFF against a whole brain ROI might reduce our sensitivity to detect a significant difference between the anterior and posterior circulation. The standardised subcortical fALFF values could also then be compared against the standardised visual cortex fALFF to determine if there is a regional difference in fALFF z-scores within the posterior circulation, to test our primary hypothesis that medullary fALFF is lower in hypertension. Mean frontal grey matter ALFF was subtracted from the voxelwise ALFF, then this was divided by the standard deviation of the frontal grey matter fALFF, to generate a map of fALFF z-scores. These were transformed to MNI space using the spatial registration matrices generated during the pre-processing stage.

Initially, we analysed rsfMRI data from a small subset of participants (141 hypertensives and 141 matched normotensive controls). The results of this analysis were used in a power calculation using the software package G*power [38]. The sample size required to achieve a power of 0.8 with a type-II error probability of 0.05 in relation to detecting a significant effect of the interaction between regional ALFF and blood pressure status was estimated to be 3461 in total. We therefore performed a largescale analysis of fALFF aiming for a minimum sample size of 3461. We limited the analysis to a subset of UK Biobank participants, whilst ensuring that the sample size was above the threshold dictated by our power calculation, because of limitations in computational processing time and data storage capacity. 2054 hypertensive rsfMRI datasets were obtained from the UK Biobank database. Propensity matching was performed to select 1724 normotensive control rsfMRI datasets in order to meet the total sample size threshold specified by the aforementioned power calculation.

Three separate two-way ANCOVAs were applied (initial analysis of small dataset 0–0.1164 Hz, largescale analysis 0–0.1164 Hz and largescale analysis 0.01–0.08 Hz) with fALFF as the dependent variable and blood pressure group (normotensive or hypertensive) and brain region as independent variables. To control family-wise error rate across the three ANCOVA tests, a Bonferroni correction was applied, and the significance level set at 0.0167. Age, BMI and sex were included as covariates. The results of validating the assumptions of parametric testing are outlined in appendix A and B.

## 3 Results

### 3.1 Initial analysis of small sample

The initial analysis of a small sample (*n* = 242) found that mean fALFF z-score across all regions of interest (brainstem, amygdala, hippocampus, hypothalamus, thalamus, insula cortex and visual cortex) in hypertensives (−0.538 (standard deviation 0.521, standard error 0.01) is significantly higher than in normotensives (−0.615 (standard deviation 0.487, standard error 0.01), F(1) = 30.03, *p* = 4.6 × 10^−8^), although the effect size is very small (partial η^2^ = 0.008). There is a significant regional difference in fALFF z-score across all participants ((F(14) =91.72, *p <* 2 × 10^−16^, partial η^2^ = 0.26). The age, sex and BMI covariates also significantly predict fALFF z-score. However, there was no statistically significant effect of the interaction between brainstem region and blood pressure group on fALFF z-score (F(14) = 0.639, *p* = 0.83, partial η^2^ = 0.002).

### 3.2 Largescale fALFF analysis 0–0.1164 Hz

As discussed in Section 2.3, the results of the initial analysis of 242 participants prompted a largescale fALFF analysis. Table 1 demonstrates that there is no significant difference in baseline demographics between the hypertensive and matched normotensive groups in the largescale analysis.

**Table 1:** Baseline characteristics (whole-brain fALFF analysis) after matching. No significant difference in age, sex or BMI between the groups.

	**Hypertensive (2054)**	**Normotensive (1724)**	**p-value**
**Age (mean)**	57.7 ± 6.7	57.5 ± 6.9	0.37
**BMI (mean)**	27.8 ± 4.0	27.6 ± 4.4	0.48
**Proportion male**	0.60	0.61	(chi-square) 0.22
**BP systolic (mmHg)**	149.0 ± 18.1	137.0 ± 17.0	<2.2 × 10^−6^
**BP diastolic (mmHg)**	87.0 ± 10.6	81.2 ± 9.7	<2.2 × 10^−6^

In the frequency range 0 and 0.1164 Hz, a two-way ANCOVA comparing blood pressure (normotensive or hypertensive) and brain region on fALFF z-score, with age, BMI and sex included as covariates, found that the mean fALFF z-score across all regions of interest (brainstem, amygdala, hippocampus, hypothalamus, thalamus, insula cortex and visual cortex) in hypertensives (−0.66, standard deviation =  0.56, standard error = 0.003) was higher than in normotensives (−0.67 (standard deviation =  0.56, standard error =  0.004), F(1) = 11.95, *p* = 0.0005), although the effect size is again extremely small (partial η^2^ = 0.0002). When the narrower frequency band of 0.01 – 0.08 Hz was considered, the mean fALFF z-score across all regions in hypertensives (−0.605, standard deviation = 0.508, standard error = 0.003) is higher than in normotensives (−0.613 (standard deviation = 0.509, standard error = 0.003)), but the Bonferroni-corrected threshold for statistical significance is not met (F(1) = 3.94, *p* = 0.047, partial η^2^ = 0.0001)..

**Fig. 3:**
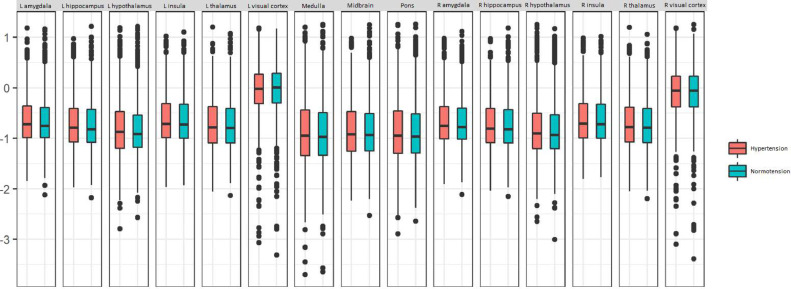
Regional fALFF z-score (0 – 0.1164 Hz) varies across the brain in both hypertensives and normotensives. There is no significant difference in the pattern of regional variation between the hypertensive and normotensive groups.

In the frequency range 0 – 0.1164 Hz there was a significant regional variation in fALFF z-score (Fig. 3, (F(14) =1126.17, *p <* 2 × 10^−16^, partial η^2^ = 0.22). Age, BMI and sex also significantly predict fALFF z-score. However, there was no statistically significant effect of the interaction between brainstem region and blood pressure group on fALFF z-score (F(14) = 0.23, *p* = 0.99, partial η^2^ = 8 × 10^−5^). The results were similar in the narrower frequency range 0.01 – 0.08 Hz, with a significant regional variation in fALFF z-score (appendix C, (F(14) = 1222.85, *p <* 2 × 10^−16^, partial η^2^ = 0.26)), but no statistically significant effect of the interaction between brainstem region and blood pressure group on fALFF z-score (F(14) = 0.29, *p* = 0.99, partial η^2^ = 0.0001).

## 4 Discussion

We have investigated whether regional fALFF differs between hypertensives and normotensives using resting state fMRI data from UK Biobank. A regional analysis was performed rather than voxelwise analysis to specifically test our hypothesis that the hypertensive medulla is differentially affected, and to mitigate the contribution of thermal noise to the fALFF. fALFF in the medulla, normalised against the frontal grey matter, was compared to several subcortical regions of interest that are part of the central autonomic network, as well as to a control region in the visual cortical grey matter. In the first instance a preliminary analysis of a small subset (*n* = 242) of participants was performed, and the results used in a power calculation to determine the sample size required to detect a significant effect of the interaction between regional fALFF and blood pressure status. Subsequently, regional fALFF between 0 and 0.1164 Hz was compared in 2054 hypertensives and 1724 normotensives. In summary, in the frequency range 0 to 0.1164 Hz, there is a statistically significant difference in mean fALFF across all ROIs between hypertensives and normotensives, but the effect size is so small as to be almost inconsequential. Furthermore, when the analysis was repeated using a narrower frequency band (0.01 – 0.08 Hz), the difference in mean fALFF did not reach the threshold for statistical significance, which suggests contributions from cardiac, respiratory and blood pressure fluctuations may be confounding fALFF in the broader frequency range. Similarly, there is a significant regional variation in fALFF (at both 0 – 0.1164 Hz and 0.01 – 0.08 Hz), but this regional variation does not differ between hypertensives and normotensives, suggesting that regional fALFF is not predictive of hypertension.

The regional variation in fALFF demonstrated in Fig. 3 is interesting, and might reflect regional differences in the contributions of physiological and thermal noise and neuronal and haemodynamic fluctuations to fALFF. The brainstem and the other subcortical grey matter structures that were included in the analysis are more caudally located and might be more susceptible to noise from cardiac, CSF and respiratory fluctuations, which might explain the difference in fALFF between these regions and the visual cortex.

This regional difference in fALFF was the same in hypertensives and normotensives, which is contrary to our primary hypothesis. However, the results do not necessarily disprove our primary hypothesis because fALFF may not be a sufficiently specific marker of cerebrovascular function. fALFF might be useful as screening tools that can be applied retrospectively in an exploratory manner to large datasets such as UK Biobank, and to guide the direction of more specific studies with targeted hypotheses. fALFF is probably less useful as a measure of specific parameters like neuronal activity or CVR, and is unlikely to be useful as a marker of cerebrovascular function in future studies of hypertension.

### 4.1 Limitations

Our aim was to use fALFF as a surrogate for CVR, and a frequency range between 0 and 0.1164 Hz was initially selected because it has been previously suggested that LFFs in this range contain information related to CVR [34]. However, the physiological basis of fALFF may not be related to a single mechanism because the biophysical origin of the BOLD signal is multifactorial. Spontaneous fluctuations in neuronal activity [39], [40], [41], [42], fluctuations in parameters including arterial O_2_ and CO_2_ [43,44], fluctuations in cerebral blood flow and cerebral blood volume driven by systemic arterial blood pressure variability modulated by cerebral autoregulation [36,45,46], as well as physiological and thermal noise [47] are all likely to contribute to low-frequency fluctuations in the BOLD signal. There is also evidence that LFFs in the frequency range of 0.01 to 0.08 Hz may be predominantly driven by spontaneous neural fluctuations [48,39]. Furthermore, Bianciardi et al. demonstrated that signal drifts at frequencies < 0.01 Hz make a significant contribution to LFFs, possibly due to head motion, slow changes in baseline physiology or changes in the baseline MRI scanner conditions [49]. Normal respiration occurs at about 0.25 Hz, which is above our cut-off frequency of 0.1164 Hz, however, it is possible in the MRI scanner environment that relaxed participants at rest might breath at a slower rate than this, potentially confounding fALFF in some cases. Furthermore, low-frequency fluctuations in heart rate [35] and systemic blood pressure [36] in the range < 0.1 Hz have been shown to correlate with fluctuations in rsfMRI signal, just below our cut-off frequency of 0.1164 Hz. It is therefore possible that cardiac pulsation or systemic blood pressure fluctuations may contribute to the LFFs between 0 and 0.1164 Hz. The results of our analysis between 0 and 0.1164 Hz were not replicated in the narrower frequency range 0.01 to 0.08 Hz, which raises the possibility that contaminating signal below 0.01 Hz and above 0.08 Hz may have adversely affected the analysis in the 0 to 0.1164 Hz frequency range.

If the selfish brain mechanism is correct, as well as alterations in cerebrovascular function, there will be an increase in neuronal activity in central autonomic network. Therefore, alterations in the BOLD signal in hypertensives might be due to neuronal as well as haemodynamic effects, further confounding any attempt to apply BOLD data to assess haemodynamic changes.

In this study only regions of the brain associated with the central autonomic network were considered. It is possible that in hypertensives, impaired CVR to other parts of the brain that were not considered might trigger an increase in sympathetic output from the central autonomic network. ICD-10 diagnostic codes were used to classify participants as hypertensive or normotensive. Some of the normotensive participants had blood pressure readings in the hypertensive range (Table 1). It is impossible to make a firm diagnosis of hypertension based on these single blood pressure readings (no ambulatory blood pressure monitoring data were available), but at least some of these apparently normotensive participants may have had undiagnosed hypertension. Furthermore, the presence of other vascular risk factors such as diabetes were not accounted for, and may have confounded the association between fALFF and hypertension.

Various other MRI techniques have been used to investigate CBF and CVR in humans. Induced hypercapnia is routinely used as a vasoactive stimulus in MRI studies of CVR [50,51]. Hypercapnia is induced by breathing a gas mixture that contains an increased concentration of CO_2_ or via a breath-hold. The response to hypercapnia can be measured using one of several methods, including transcranial Doppler ultrasound measurement of the change in diameter of the middle cerebral artery [52], the BOLD signal response to hypercapnia [53], or by directly measuring CBF using ASL [54]. These may all be more robust methods of measuring CVR, but unfortunately they are not typically present in large biomedical databases. They are also technically challenging to acquire compared to rsfMRI data. A surrogate marker of vascular reactivity such as fALFF is therefore potentially valuable as a tool to explore large datasets before applying a more robust measure of CVR in a prospective study. This was a retrospective study using data from the UK Biobank database. Categorisation of participants as hypertensive was made based on ICD-10 diagnoses derived from clinical records, but we were unable to fully ascertain the medication history of the participants, and therefore could not study the impact of antihypertensive medication.

## 5 Conclusion

In this study we investigated the association between fALFF and hypertension in the context of the selfish brain mechanism, a proposed aetiological mechanism for hypertension. Mean fALFF in the frequency range 0 – 0.1164 Hz across the brainstem, amygdala, hippocampus, hypothalamus, thalamus, insula cortex and visual cortex, calculated from resting state BOLD signal, is significantly higher in hypertensives, but the effect size is extremely small, and this finding is not replicated in the narrower frequency range of 0.01 – 0.08 Hz. There is a significant regional variation in fALFF across the brain but there was no association between regional fALFF and hypertension. fALFF in the medulla does not seem to have a specific association with hypertension. Prospective longitudinal studies of cerebral haemodynamics in hypertensives and normotensives are required to further investigate the selfish brain mechanism. fALFF is unlikely to be useful as a specific distinguishing marker of cerebrovascular reactivity in the context of hypertension, but might be useful as a general measure of cerebrovascular function in other cerebrovascular pathologies.

## CRediT authorship contribution statement

**Owen Bleddyn Woodward:** Conceptualization, Formal analysis, Investigation, Methodology, Project administration, Software, Validation, Visualization, Writing – original draft, Writing – review & editing. **Ian Driver:** Conceptualization, Formal analysis, Investigation, Methodology, Project administration, Resources, Supervision, Validation, Visualization, Writing – review & editing. **Emma Hart:** Supervision, Writing – review & editing. **Richard Wise:** Conceptualization, Investigation, Methodology, Project administration, Supervision, Validation, Writing – review & editing.

## Declaration of Competing Interest

The authors declare that they have no known competing financial interests or personal relationships that could have appeared to influence the work reported in this paper.

## Figures and Tables

**Fig. A1:**
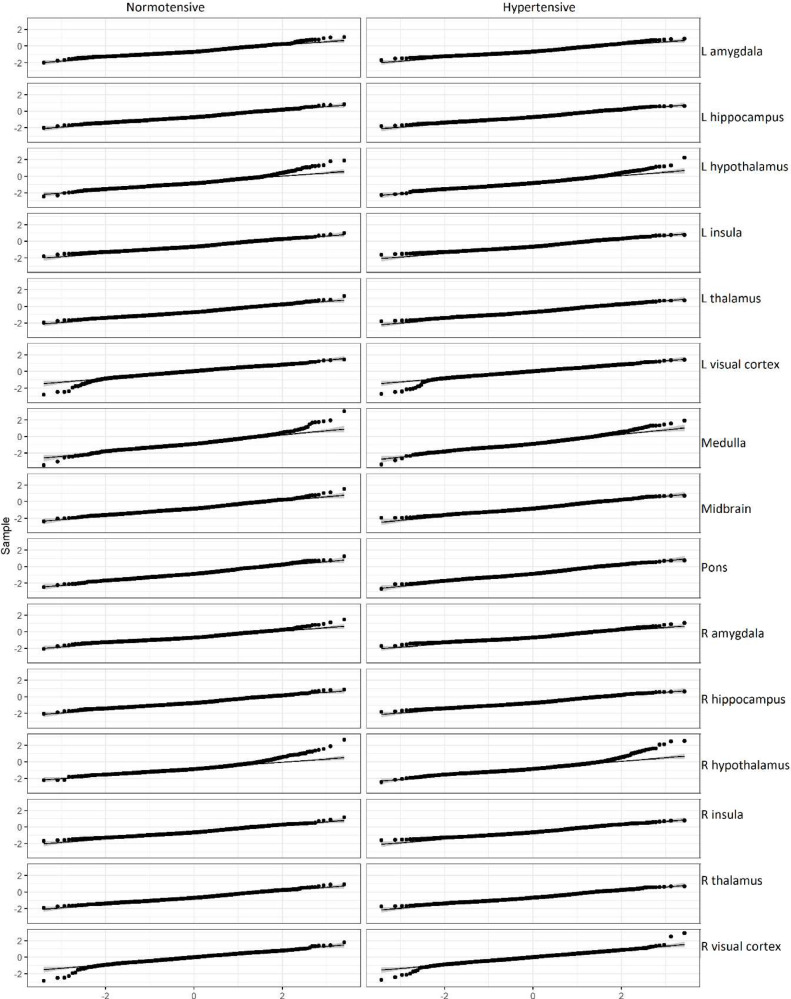
Q-Q plots of fALFF grouped by region and blood pressure status reveals that there is some deviation from normality in the distribution of fALFF values in some of the groups. However, the majority of fALFF values conform to a normal distribution. ANCOVA is robust against deviations from normality for large samples [55].

**Table B1:** Summary of mean and standard deviation of fALFF z-scores for all regions in normotensives and hypertensives. The ratio of the largest to smallest standard deviation is 1.46 [56]. The data is therefore homoscedastic. Regardless, ANCOVA is robust against violations of the assumption of homogeneity of variance as long as group sizes are equal (equal group sizes being defined as: ratio of the largest to smallest group < 1.5) [57].

**BP group**	**Region**	**Mean fALFF z-score**	**SD fALFF z-score**
Normotension	L amygdala	−0.622	0.394
Hypertension	L amygdala	−0.607	0.395
Normotension	L hippocampus	−0.687	0.404
Hypertension	L_hippocampus	−0.677	0.409
Normotension	L hypothalamus	−0.787	0.474
Hypertension	L hypothalamus	−0.774	0.478
Normotension	L insula	−0.599	0.404
Hypertension	L insula	−0.589	0.419
Normotension	L thalamus	−0.659	0.419
Hypertension	L thalamus	−0.652	0.422
Normotension	L visual cortex	0.022	0.45
Hypertension	L visual cortex	0.006	0.439
Normotension	Medulla	−0.824	0.573
Hypertension	Medulla	−0.812	0.577
Normotension	Midbrain	−0.788	0.47
Hypertension	Midbrain	−0.785	0.469
Normotension	Pons	−0.813	0.493
Hypertension	Pons	−0.808	0.501
Normotension	R amygdala	−0.643	0.396
Hypertension	R amygdala	−0.632	0.402
Normotension	R hippocampus	−0.691	0.408
Hypertension	R hippocampus	−0.684	0.412
Normotension	R hypothalamus	−0.779	0.501
Hypertension	R hypothalamus	−0.766	0.518
Normotension	R insula	−0.601	0.404
Hypertension	R insula	−0.593	0.415
Normotension	R thalamus	−0.654	0.414
Hypertension	R thalamus	−0.646	0.418
Normotension	R visual cortex	−0.027	0.458
Hypertension	R visual cortex	−0.011	0.461

**Table:** 

**Baseline characteristics (regional analysis) after matching. No significant difference in age, sex or BMI between the groups.**			
	**Hypertensive (*n*** **=** **141)**	**Normotensive (*n*** **=** **141)**	**p-value**
**Age (mean)**	54.7 ± 7.6	54.6 ± 7.5	0.85
**BMI (mean)**	28.5 ± 4.3	28.4 ± 4.6	0.66
**Proportion male**	0.62	0.61	(chi-square) 0.60
**BP systolic (mmHg)**	147.6 ± 18.8	137.4 ± 16.4	<6.9 × 10^−6^
**BP diastolic (mmHg)**	88.4 ± 17.5	82.9 ± 9.4	<2.4 × 10^−5^

## Appendix A

See Fig. A1

## Appendix B

See Table B1

## Appendix C

The demographics of the hypertensive and normotensive groups for the initial analysis of 242 patients are summarised in the table below. After propensity matching there was no significant difference in age, sex, or BMI between the two groups in either of the analyses.

Regional fALFF z-score (0.01 – 0.08 Hz) varies across the brain in both hypertensives and normotensives. There is no significant difference in the pattern of regional variation between the hypertensive and normotensive groups.Similar result to the analysis between 0 – 0.1164 Hz.
